# Insights into the Antimicrobial, Antioxidant, Anti-SARS-CoV-2 and Cytotoxic Activities of *Pistacia lentiscus* Bark and Phytochemical Profile; In Silico and In Vitro Study

**DOI:** 10.3390/antiox11050930

**Published:** 2022-05-09

**Authors:** Samy Selim, Mohammed S. Almuhayawi, Mohanned T. Alharbi, Soad K. Al Jaouni, Afaf Alharthi, Basel A. Abdel-Wahab, Mervat A. R. Ibrahim, Amnah Mohammed Alsuhaibani, Mona Warrad, Khaled Rashed

**Affiliations:** 1 Department of Clinical Laboratory Sciences, College of Applied Medical Sciences, Jouf University, Sakaka 72388, Saudi Arabia; 2 Department of Medical Microbiology and Parasitology, Faculty of Medicine, King Abdulaziz University, Jeddah 21589, Saudi Arabia; 3 Department of Medical Microbiology and Parasitology, Faculty of Medicine, University of Jeddah, Jeddah 23218, Saudi Arabia; mtalharbi@uj.edu.sa; 4 Department of Hematology/Oncology, Yousef Abdulatif Jameel Scientific Chair of Prophetic Medicine Application, Faculty of Medicine, King Abdulaziz University, Jeddah 21589, Saudi Arabia; skaljaouni@kau.edu.sa; 5 Department of Clinical Laboratory Sciences, College of Applied Medical Sciences, Taif University, Taif 21944, Saudi Arabia; a.awwadh@tu.edu.sa; 6 Department of Medical Pharmacology, College of Medicine, Assiut University, Assiut 71515, Egypt; basel_post@msn.com; 7 Department of Pharmacology, College of Pharmacy, Najran University, Najran 55461, Saudi Arabia; 8 Biochemistry Department, Faculty of Agriculture, Ain Shams University, Cairo 11566, Egypt; mervat_ibrahim@agr.asu.edu.eg; 9 Department of Physical Sport Science, College of Education, Princess Nourah bint Abdulrahman University, Riyadh 11671, Saudi Arabia; amalsuhaibani@pnu.edu.sa; 10 Department of Clinical Laboratory Sciences, College of Applied Medical Sciences at Al-Quriat, Jouf University, Al Qurayyat 77425, Saudi Arabia; mfwarrad@ju.edu.sa; 11 Pharmacognosy Department, National Research Centre, 33El Bohouth Str., Dokki, Giza 12622, Egypt

**Keywords:** *Pistacia lentiscus*, foodborne control, antioxidant, antibacterial activity, antifungal activity, antiviral activity, SARS-CoV-2, 3CL-protease, molecular modelling, cytotoxic activity, phytoconstituents

## Abstract

Foodborne infections and antibiotic resistance pose a serious threat to public health and must be addressed urgently. *Pistacia lentiscus* is a wild-growing shrub and has been utilized for medicinal applications as well as for culinary purposes. The antibacterial and antioxidant activities of P. lentiscus bark in vitro, as well as the phytochemical composition, are the focus of this inquiry. The bark extract of *P. lentiscus* showed significant antimicrobial activity in experiments on bacteria and yeast isolated from human and food sources. The exposure time for the complete inhibition of cell viability of *P. aeruginosa* in the extracts was found to be 5% at 15 min. Phytochemical inquiry of the methanol extract demonstrates the existence of carbohydrates, flavonoids, tannins, coumarins, triterpenes, and alkaloids. Deep phytochemical exploration led to the identification of methyl gallate, gallic acid, kaempferol, quercetin, kaempferol 3-O-α-rhamnoside, kaempferol 3-O-β-glucoside, and Quercetin-3-O-β-glucoside. When tested using the DPPH assay, the methanol extracts of *P. lentiscus* bark demonstrated a high free radical scavenging efficiency. Further, we have performed a molecular modelling study which revealed that the extract of *P. lentiscus* bark could be a beneficial source for novel flavonoid glycosides inhibitors against SARS-CoV-2 infection. Taken together, this study highlights the *Pistacia lentiscus* bark methanol extract as a promising antimicrobial and antiviral agent.

## 1. Introduction

There is a growing need for novel compounds to replace antibiotics because of increasing resistance to the treatments and their unwanted side effects [1]. There has also been an increase in the use of NSAIDs and even steroids as anti-inflammatory agents, despite the fact that these medications typically have severe adverse effects [2]. In light of these findings, a growing number of researchers are turning to plants for drug discovery in an effort to prove scientifically that they are popular cures for a variety of ailments [3].

Examples of compounds derived from natural plants include statins, tubulin-binding anticancer medicines, and certain immunosuppressants [4,5]. Traditional folk medicine in the Mediterranean region has long employed extracts and oils obtained from plants such as *Malva* spp., *Thymbra capitate* (L.) Cav., and *Olea europaea* L. [6].

Several chemical classes influence the biological activity of essential oils and polyphenols derived from plants and herbs [7,8]. In this context, the terpenes and terpenoids found in essential oils are promising agents in preventing and treating diseases, including cancer [9]. Terpenes and terpenoids have been studied in the context of human disease for many decades, with positive results in inflammatory diseases, tumorigenesis, and neurodegeneration being observed in cell and animal models [10]. These studies have suggested that terpenes and terpenoids may be used as chemopreventive and therapeutic agents [11]. In addition, polyphenols have been shown to possess a variety of beneficial qualities, with compounds such as tannins, flavonoids, and lignin-carbohydrate complexes all being associated with significant antibacterial, anti-inflammatory, and antioxidant activities, respectively [12].

Pistacia (Anacardiaceae) is a genus of nine species and five subspecies. The genus contains dioecious evergreen, deciduous shrubs, or small trees that are classified as xerophytic species [13]. *Pistacia lentiscus* L. (often referred to as lentisk) is a highly distinctive component of Mediterranean ecosystems. It is an evergreen shrub that grows to 2–3 m [14]. In this context, *Pistacia lentiscus* L. is a wild-growing plant full of polyphenols and terpenoids [15]. Despite the fact that there has been much research into the leaves, fruits, natural aromatic resin, and its clinical applications, there is still much more research on tree bark extracts that have not yet been compiled into a comprehensive report. Currently, there is widespread scientific interest in the edible and non-edible sections of *Pistacia lentiscus*, since several types of research have highlighted the possible value of these components in the prevention and treatment of inflammation and infections [16]. Aside from that, the high concentration of polyphenols present in the extracts makes them appealing as anti-cancer and anti-degenerative disease agents and nutraceuticals in human health [17].

Given the foregoing concerns, the goal of this research was to test the biological properties of Pistacia lentiscus bark extracts. In the current study, we have inspected phytochemical constituents of the *P. lentiscus* bark extract and in vitro explored the antimicrobial activity against food and clinical isolates. Further, we have in silico examined the efficacy of *P. lentiscus* bark extract as a possible inhibitor against SARS-CoV-2 infection.

## 2. Materials and Methods

### 2.1. Plant Gathering and Identification

*P. lentiscus* bark was collected from Al-Zohiriya garden, Giza, Egypt. A voucher specimen was deposited in the herbarium at Al-Zohiriya garden, Giza, Egypt. The plants were identified by Mrs. Tereez Labib, director of the Orman botanical garden, Giza, Egypt, and consultant of Plant Taxonomy at the Ministry of Agriculture, and Dr. Mohammed El-Gebaly, National Research Centre (NRC).

### 2.2. Extraction of P. lentiscus Bark

The bark of *P. lentiscus* (850 g) was extracted five times with 80% methanol at ambient temperature to provide a complete extract. The obtained mixture was filtered, and the solvent was removed under decreased pressure to afford 66.5 g of dry extract [18].

### 2.3. Antimicrobial Assessments

#### 2.3.1. Yeast and Bacterial Strains

The yeast and bacterial strains utilized in the current study were isolated from food and human samples that belong to the microbiology laboratory, Department of Clinical Laboratory Sciences, College of Applied Medical Sciences, Jouf University, Saudi Arabia. Yeast and bacterial strains were injected onto Petri dishes using Sabouraud dextrose agar and nutrient agar media, respectively.

#### 2.3.2. Disc Diffusion Assay

The adapted Kirby–Bauer disc diffusion method [19] was employed to conduct the agar diffusion experiment. In 3 mL of 0.9% NaCl solution, 1 mL of the liquid culture of each test organism was suspended. Millipore filters were used to sterilize the 30 mg/mL stock solution of methanol extract, which was then diluted in 10% dimethylsulfoxide (DMSO). Tests for antimicrobial activity were then conducted using 100 μL of bacteria (10^8^ cfu/mL) and yeast (10^6^ cfu/mL) suspensions, distributed over the medium. A total of 100 μg of the extract was then added to coat the discs which were 6 mm in diameter, and subsequently, the discs were placed on inoculated agar. The solvent applied to the extract stock solutions was utilized to produce negative controls. For clinical and foodborne bacterial strains, plates were incubated at 37 °C for 24 h, and for yeast isolates plates were incubated for 48 h. The zone of inhibition toward the pathogens under investigation was employed to measure antimicrobial potency. Ampicillin, gentamicin and amphotericin are standard antibiotics used as a reference for antimicrobial activity.

### 2.4. Cell Toxicity Assay for Pseudomonas aeruginosa

Each of the bacterial suspensions containing 10^6^ cfu/mL of *Pseudomonas aeruginosa* was inoculated with 0.25, 0.5, and 1% concentration of *P. lentiscus* bark methanol extract in 10 mL MHB. The samples were kept at 37 °C, and the cell viability was examined at 0, 5, 10, 15, 20, 40, 60, and 120 min time intervals. Each concentration was tested in triplets. The cell viability was examined as follows: Each treatment (100 μL) was diluted and coated on the surface of MHB agar. After incubation at 37 °C for 24 h, the number of colonies were counted. The same experimental conditions were set as mentioned above without extract as the control set [20].

### 2.5. General Experimental Procedures

The ultraviolet analysis (UV/VIS) was carried out employing a Shimadzu UV-visible recording spectrophotometer model-UV 240 (NRC, Cairo, Egypt). The nuclear magnetic resonance (^1^H- and ^13^C-NMR) analysis was performed via Varian Unity Inova (IET, Urbana, IL, USA). Mass spectroscopy was performed via Finnigan MAT SSQ 7000, 70 ev (Adaptas Solutions, Palmer, MA, USA). Flash column chromatography was employed using a Sephadex LH-20 (Sigma-Aldrich, Taufkirchen, Germany), obtained from Pharmacia Fine Chemicals, and silica gel (0.063–0.200 mm). Paper Chromatography (PC) was performed on Whatman No.1 sheets (Whatman Led. Maid Stone, Kent, UK) to quantify flavonoids and sugars in the extract. Thin-layer chromatography (TLC) analysis was performed on F_254_ plates. The solvent mixtures for separation were applied (*n*-butanol: acetic acid: water 4:1:5 upper phase, 15% acetic acid: water: glacial acetic acid: 85:15).

### 2.6. Isolation of Compounds from P. lentiscus Bark Methanol Extract

Using silica gel column chromatography, 25 g of the extract was progressively eluted with *n*-hexane (300 mL), dichloromethane (400 mL), ethyl acetate (400 mL), and methanol (500 mL). It was necessary to collect a total of 140 fractions of 100 mL/each. Four fractions were created by combining the fractions that exhibited comparable PC results in Butanol–Acetic acid–Water 4:1:5 and 15% acetic acid (I, II, III, and IV). Compound **1** was obtained by elution with dichloromethane: *n*-hexane (50:50) from fraction I (2.45 g), whereas compound **2** was obtained by elution with dichloromethane: *n*-hexane (80:20) from fraction I (2.45 g). Extraction of the silica gel sub-column generated chemical **3**, and the subsequent elution with ethyl acetate solvent provided the final product, compound **4**. Ethyl acetate: methanol (90:10) produced compound **5**, while fraction III’s ethyl acetate: methanol (80:20) produced compound **6** (2.75 g). Fraction IV produced compound **7** and compound **8** by elution with 75:25 mixtures of ethyl anhydride and methanol, respectively (3.22 g). Methanol and distilled water were used to purify all the identified chemicals on a Sephadex LH–20 column [21].

### 2.7. Analysis of Flavonoid Glycosides by Acid Hydrolysis

Flavonoid glycosides 5, 6, 7, and 8 (5 mg) were heated in a solution of 10% HCl. After 5 h, the mixture was extracted with ethyl acetate to afford crude aglycones. The crude mixture was separated and analyzed by TLC utilizing reference standards. The aqueous layer was analyzed by paper chromatography Whatman No. 1 sheets in the presence of reference standards employing a solvent system (*n*-BuOH-AcOH-H_2_O 4:1:5 upper layer) to provide the sugar content of the flavonoid [22].

### 2.8. Analysis of Antioxidant Capability

#### 2.8.1. Assessment of Antioxidant Activity

This method is based on the ability of the tested extract to convert Mo (VI)–Mo (V) and to form the green phosphate/Mo (V) complex at acidic pH. A reagent solution of 3 mL (28 mM sodium phosphate, 0.6 M sulphuric acid, and 4 mM ammonium molybdate) was added to the 0.1 mL of extract used for the experiment. The resulting reaction mixture was allowed to incubate at 95 °C for 90 min. After the reaction solution was cooled to ambient temperature, the solution absorbance was assessed at 695 nm against a blank. This activity was measured in ascorbic acid equivalents [23].

#### 2.8.2. DPPH Scavenger Activity

When the purple DPPH solution in methanol is bleached, the capacity of the extracts or pure chemicals to donate hydrogen atoms or electrons may be determined. Due to being an experiment that relies on stable radicals, this spectrophotometric assay makes use of DPPH as its reagent. The methanolic extract was mixed with a DPPH solution (0.004% (*w*/*v*) methanol), and 50 microliters of each concentration was added to the solution. After the mixture was incubated for 30 min at ambient temperature, the absorbance was assessed at 517 nm against a blank. The following formula was applied to obtain the percentage of free radical DPPH inhibition (I%):

I% = (A_control_ − A_sample_/A_control_) × 100 (1)

A_control_ represents the absorbance of the control sample (without methanolic extract sample), and A_sample_ represents the absorbance of the methanolic extract sample. IC_50_ were considered from the regression equation and based on the extract concentration, and the ratio of free radical formation inhibition, the percentage inhibition of the DPPH was evaluated. Reference antioxidant standards, butylated hydroxyanisole (BHA), and L-ascorbic acid have been utilized as reference controls [24,25].

### 2.9. In Silico Molecular Modelling Study

The binding of the different components of the extracts (1–5) was explored toward the binding pocket of 3-chymotrypsin-Like (3CL-) protease protein of SARS-CoV-2 virus by computational study employing Molecular Operating Environment software (MOE, 2015.10). Several 3D X-ray crystal-structures for 3CL-preotease with different substrates and inhibitors are available in the protein data bank (PDB) [26]. To deeply investigate the potency of compounds **1**–**5** to target 3CL-protease activity, we have performed molecular docking studies using the recent reported crystal structure of 3CL-protease co-crystallized with baicalein (PDB code: 6m2n), since compounds **1**–**5** have similar scaffolds to baicalein [27]. The 2D structures of compounds **1**–**5** were obtained using the ChemDraw Professional (cds.15.1) program (PerkinElmer, Waltham, MA, USA) and converted to a 3D format using Discovery Studio software (Dassault Systèmes, Vélizy-Villacoublay, France). The 3D structure of the 3CL-protease protein (PDB code: 6m2n) was acquired from the PDB website (http://www.rcsb.org/pdb, 1 March 2022) The 3D structure of the 3CL-protease protein was protonated, and the water and extra chains were removed using the default mode in the MOE program. Default Conf Search module and MMFF94x force field were applied to express the charges and to optimize the geometry. Next, the docking protocol was validated by re-docking baicalein into the binding pocket of 3CL-protease applying the London dG scoring function and Triangle Matcher placement method [28,29,30,31,32,33]. The acquired poses were evaluated based on the reported binding mode to confirm the similarity in main interactions. Subsequently, the validated method was applied to explore and dock compounds **3**–**6** into the active site of 3CL-protease. The obtained data were evaluated to get the poses with the highest binding affinity score.

### 2.10. Statistical Analysis

Standard deviation and the student t-test were used to determine the statistical significance of differences between experiments. Only the probability *p* ≤ 0.05% was considered symbolic of statistical impact.

## 3. Results and Discussion

When it comes to Mediterranean scrubland (maquis), *P. lentiscus* is one of the most common shrubs to be found [34]. Eco-friendly *P. lentiscus* is an evergreen plant. Genotype and secondary metabolite richness are influenced by challenging growing circumstances, dryness, and a warm climate [35]. Pistacia lentiscus has been used in a variety of ways over the ages. Nuragic civilization (1800–238 BCE): was used by Sardinian people as a popular medicine and for lighting lamps in their homes, or as votive oil for religious purposes [36].

### 3.1. Phytochemical Properties of P. lentiscus Bark Extract

**Figure 1:**
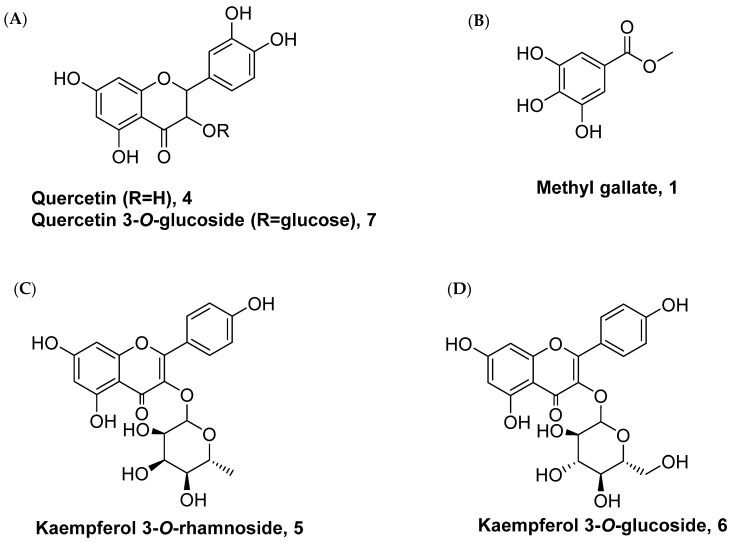
Chemical structure of the compounds isolated from *P. lentiscus* bark.

We first investigated the phytochemical properties of the methanolic extract of *P. lentiscus* bark. The results showed that the methanolic extract comprised of triterpenes, carbohydrates, tannins, alkaloids, and flavonoids (Table 1). Further analysis of the extract to identify the major constituents revealed that the methanolic extract of *P. lentiscus* bark includes gallic acid, quercetin (Figure 1A), methyl gallate (Figure 1B), kaempferol, kaempferol 3-O-α-rhamnoside (Figure 1C), kaempferol 3-O-β-glucoside (Figure 1D), and Quercetin-3-O-β-glucoside. The chemical structures of selected isolated compounds are shown in Figure 1. Purification of the methanolic extract by chromatographic methods led to the isolation of different compounds. These compounds were exhibited on thin-layer chromatography (TLC) as dark spots under short UV light, and as violet spots after treatment with vanillin sulphuric acid at 110 °C for 5 min. Compound **4** exhibited a yellow spot and showed a fluorescent yellow color spot after treatment with AlCl_3_. The spectroscopic spectra revealed its structures as quercetin (Supplementary Information) [37]. Compound **5** appeared as a yellow spot after treatment with ammonia and AlCl_3_ and exhibited under UV light as a deep purple spot. Acidic hydrolysis of compound **6** afforded quercetin (aglycone) and rhamnose (sugar moiety). The analytical spectra revealed the chemical structure as quercetin 3-O-α-rhamnoside [38]. Compound **6** (quercetin 3-O-β-glucoside) showed under UV light as a deep purple spot and appeared as a yellow colour spot after being subjected to ammonia vapour, while exhibiting a bright yellow colour spot after being treated with AlCl_3._ Acidic hydrolysis of compound **6** provided quercetin (aglycone) and glucose (sugar moiety). Finally, spectroscopic data demonstrated compound **6** as quercetin 3-O-β-glucoside [39]. Water and alcoholic extracts are the common methods of extraction for the *P. lentiscus* leaf, seeds, and bark. Furthermore, hydro-distillation using Clevenger-type devices is mainly used to extract the oil [3]. Each type of extraction will give different organoleptic profiles and chemical compositions. Many reports found that the antibacterial capacity, for example, is said to be higher in a material solvent extract than in a hydro-distilled method [40]. It has been found that GC/MS and HPLC are the most effective methods for quantifying phytochemical content in the extracts [40]. *P. lentiscus* is made up of a mix of terpenes and terpenoids, mostly monoterpenes and sesquiterpenes, making the plant smell and taste unique [15,41]. In addition to environmental conditions, harvesting season, and plant part, the composition of the extracts must be taken into account when explaining the changes in chemistry [36]. The extract can be categorised into various chemotypes based on the dominant fractions of monoterpenes and oxygenated sesquiterpenes [34]. As long as harvesting locations are spread around the country, it is possible to have multiple chemotypes in the same spot at the same time [42].

### 3.2. Antimicrobial Activity of P. lentiscus Bark Extract

We have next evaluated the antimicrobial activity of methanolic extracts of *P. lentiscus* bark. As indicated in Table 2, the methanol extract from the *P. lentiscus* bark has antibacterial properties against a variety of micro-organisms in vitro. The extract demonstrated substantial antimicrobial activity with width inhibition zones ranging from 10 to 25 mm for all studied micro-organisms. Comparing the susceptibility of the extract to different food and clinical pathogens, *Serratia marcescens* appears to be more resistant to *P. lentiscus* extract. Moreover, our results found that the *P. lentiscus* extract has a greater antibacterial effect on Gram-positive bacteria than Gram-negative bacteria. *P. lentiscus* bark extract has been shown to have antimicrobial activity in several investigations, which aimed to scientifically explain their widespread use in treating infectious disorders. *P. lentiscus* terpenoids, which have been shown to prevent the growth of drug-resistant micro-organisms that are difficult to treat even with conventional antibiotics, served as the starting point for the research. The superior resistance among Gram-negative bacteria could be attributed to the phospholipidic membrane, which is almost impermeable to lipophilic compounds [43]. When this barrier is not present in Gram-positive bacteria, ion permeability and leakage of key intracellular components might occur, leading to either an impairment of the bacterium enzyme or an increase in ion permeability and leaking of critical intracellular components [44].

The antibacterial properties of *P. lentiscus* bark methanol extract might be attributed to the presence of triterpenes and flavonoids. Several studies have shown the antibacterial properties of these components [45,46]. Phenolics, flavonoids, and terpenoids may be accountable for the antibacterial properties of *P. lentiscus* bark [47].

The presence of phenolic chemicals in the plant extract is primarily responsible for its antibacterial effect [48]. As represented in the literature, the sequestered complexes from the *P. lentiscus* bark methanolic extract demonstrated potent antimicrobial activity [49,50,51]. Examination of the phytochemical properties of the extract revealed that flavonoids are the major constituents of the extract, including quercetin-3-O-glycoside, luteolin-7,3’-O-diglycoside apigenin, kaempferol-3-O-glycoside, and luteolin-7-O-glycoside. Among identified flavonoids, it was realized that the main antibacterial activity could be attributed to quercetin-3-O-glucoside. Nevertheless, the natural mixture of kaempferol-3-O-glycoside, quercetin-3-O-glycoside, and apigenin afforded the pre-eminent inhibitory attention. There are also several classes of flavonoids that showed potent influence on the antibacterial action [52].

### 3.3. Cell Toxicity Assay for Pseudomonas aeruginosa

In comparing microbial sensitivity to *P. lentiscus* bark methanol extract, *Pseudomonas aeruginosa* appears more susceptible than other reported infectious pathogens. Furthermore, the effect on the cell capabilities of *P. aeruginosa* confirmed the acquaintance of extract at 0.25, 0.5, and 1% concentrations. *P. lentiscus* bark methanol extract had a potential antibacterial effect on the capabilities of *P. aeruginosa*. The contact times for the comprehensive embarrassment of cell viability of *P. aeruginosa* in extracts was found to be 5% at 15 min. Infections caused by the bacterium Pseudomonas aeruginosa are common in severely unwell individuals. Additionally, the mortality rate linked with *P. aeruginosa* infection is substantially higher than that associated with other bacteria [53]. Different strains of *P. aeruginosa* have recently been classified as invasive or cytotoxic [54]. This cytotoxicity could be related to quorum sensing [53]. In this study, the disruption of membrane integrity was suggested to be the mode of action of the *P. lentiscus* bark extract. The extracts may have penetrated the Gram-negative *P. aeruginosa* outer membrane by pre-disruption of the membrane [55].

### 3.4. Assessment of Antioxidant Activity of P. lentiscus Bark Extract

The methanolic extract of *P. lentiscus* bark demonstrated a moderate total antioxidant activity compared to that of ascorbic acid. The antioxidant capacity was assessed utilizing the revised equation based on the concentration versus optical density of the investigated compound and the reference control. The extraction process was performed by employing methanol to afford 54.34 ± 2.3 µg of dried *P. lentiscus* bark. Table 3 shows the antioxidant activity of the methanol extract of *P. lentiscus* bark by investigating DPPH radical scavenging activity. As the IC_50_ value decreases, this indicates a high antioxidant activity. In our investigations, the reference controls (BHA and ascorbic acid) demonstrated values 3.1 and 3.7 times greater than the *P. lentiscus* bark methanolic extract, respectively. Notably, the methanolic extract demonstrated a considerable antioxidant activity at low concentrations. The DPPH free radical assay has been extensively employed as a tool to assess antioxidant activity by evaluating free radical-scavenging activity [56]. The reaction of the antioxidant with DPPH involves neutralization of the free radical character by transferring hydrogen atoms or electrons to DPPH [57]. The key characteristics of phenolic compounds as free radical scavengers and antimicrobials have been accentuated in several studies [58,59]. Based on Borchers et al. [60], plant extracts could be more valuable than isolated ingredients since the presence of other complexes in the crude extracts could modify the possessions of isolated bioactive constituents. According to research, the polyphenol content of extracts is responsible for this ability, which prevents ROS formation by interacting directly with particular molecules and alters the Keap1-Nrf2/ARE pathway [14]. Polyphenols degrade the Keap1 protein and regulate the Nrf2-related pathway as part of this effective oxidation-reduction defence system [61]. In proportion to the peak of hydroxyl groups, the aqueous formulations strongly reduced lipid peroxidation. The mechanism was explained by the scavenging of peroxyl radicals by the *P. lentiscus* bark extracts [3].

### 3.5. In Silico Molecular Modelling Study

**Figure 2:**
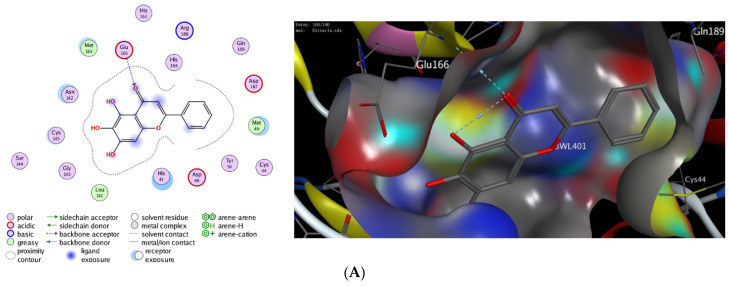

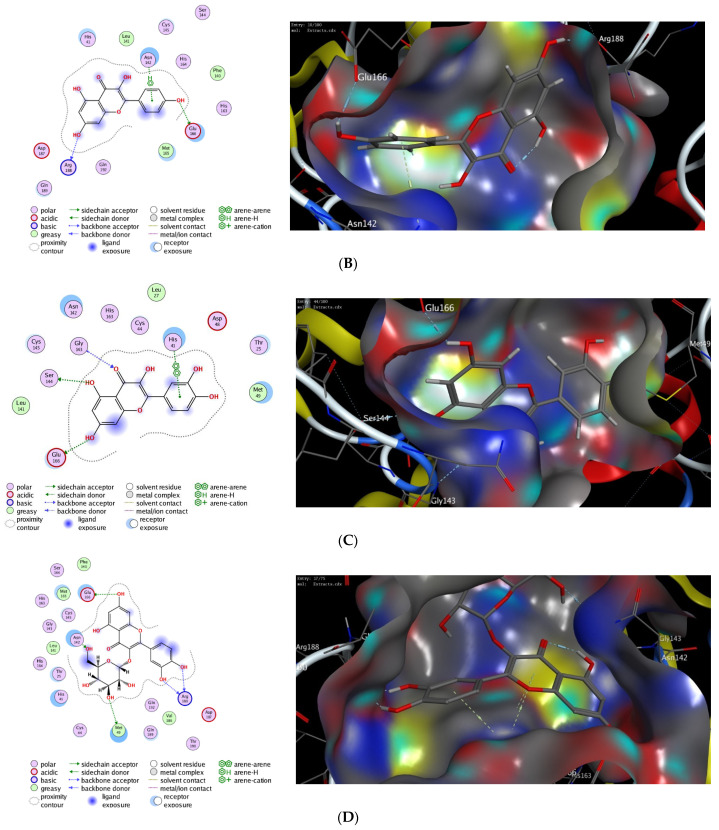

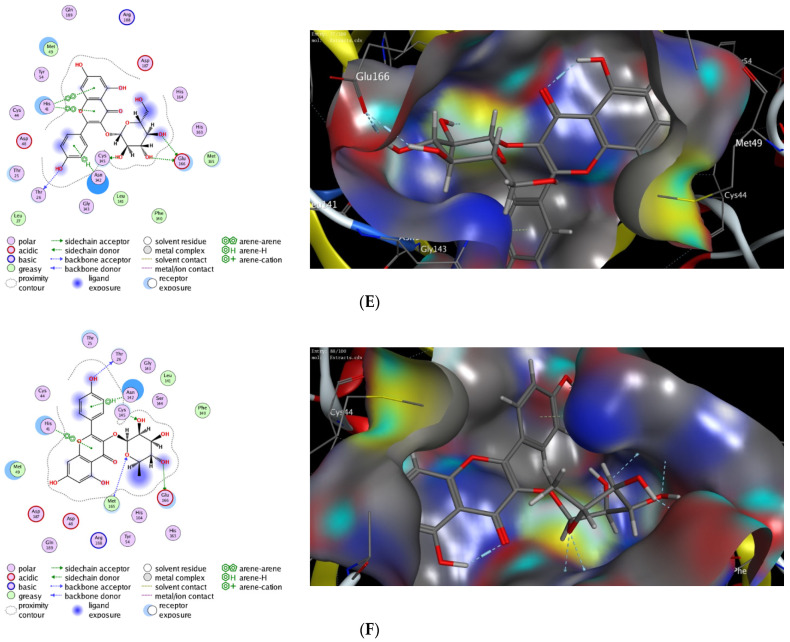
The 2D and 3D molecular docking interactions of Baicalein (**A**), Kaempferol (**B**), Quercetin (**C**), Quercetin 3-O-β-glucoside (**D**), Kaempferol 3-O-β-glucoside (**E**), and Kaempferol 3-O-*α*-rhamnoside (**F**), (gray in 3D interactions) with 3CL-protease protein (PDB code: 6m2n). The hydrogen bonds are illustrated as dotted blue arrows (C atoms are colored gray, S yellow, and O red).

Computational molecular modeling techniques demonstrated the ability to explore the efficacy of the binding affinity of bioactive molecules toward a targeted protein [26,62,63,64,65,66]. In this study, we showed that the extract of *P. lentiscus* bark possesses potent antioxidant and antimicrobial activities. To further explore the activity of this extract to act against SARS-CoV-2 infection, we have investigated the potency of the identified compounds **3**–**6** as inhibitors against the 3CL-protease activity. Toward this aim, we have performed in silico molecular docking studies to investigate the binding affinity of compounds **1**–**5** toward the binding pocket of 3CL-protease protein. There are several 3D X-ray crystal structures for 3CL-protease protein with different substrates and inhibitors [26]. In our study, we have performed our investigations using the recently reported 3D structure of 3CL-protease co-crystalized with baicalein (PDB code: *6m2n*), since compounds **1**–**5** have similar flavone scaffolds to baicalein (Table 4) [27]. First, we have examined our docking protocol by re-docking baicalein to the binding site of 3CL-protein in order to validate our docking method. As shown in Figure 2, baicalein demonstrated the main interactions which have been previously reported in the original crystal structure. Indeed, baicalein displayed the ability to bind to the active pocket of 3CL-protease by forming two main hydrophilic interactions with Glu166 and Gly143 amino acid residues [27]. Subsequently, we have successfully applied the validated docking method to examine the binding affinity of compounds **1**–**5**. As indicated in Table 4, all compounds showed the ability to bind to the active site of 3CL-protease protein with high binding affinity scores. The binding of the compounds was thermodynamically favorable, as demonstrated by the negative binding score values. Furthermore, all the compounds demonstrated the ability to form extra hydrogen bonding with several amino acid residues in the binding pocket of 3CL-protease (Figure 2). As shown in Figure 2, Quercetin alone displayed the ability to bind to the active site of 3CL-protease via binding of the benzopyran-4-one scaffold to three amino acid residues (Glu166, Ser144, Gly143). In this case, the phenolic hydroxyl groups in the 2-phenyl moiety have not participated in the binding of the molecule. Nevertheless, it formed a hydrophobic interaction (H-arene interaction) with the Asn142 residue. Introducing a glucosyl moiety to the Quercetin at the 3-position of the benzopyran-4-one resulted in a significant increase in the binding affinity of the compound (Figure 2C). Indeed, Quercetin 3-O-β -glucoside revealed a set of seven hydrogen bonding interactions with the amino acid residues in the 3CL-protease binding pocket. In that case, the sugar part played an important role in the binding affinity of the compound by binding to four amino acid residues (Asn142, Met44, Met49, His41), while the 2-phenyl moiety formed, through the phenolic groups, two hydrogen bonding with Arg188 amino acid residue.

On the other hand, Kaempferol revealed only two hydrophilic interactions through the phenolic groups in the 2-phenyl and benzopyran-4-one moieties with Glu166 and Arg188 amino acid residues, respectively. Additionally, Kaempferol displayed a set of hydrophilic interactions with the greasy amino acid residues (Leu141, Met165, Phe140) [67]. Similarly, introducing a glucosyl group at 3-position in the benzopyran-4-one moiety, Kaempferol 3-O-β-glucoside, improved the binding affinity of the compound (S = −9.24 kcal/mol). As indicated in (Figure 2E), the glucosyl group formed three hydrogen bonding with Glu166 and Cys145 amino acid residues. In addition, a set of different hydrophobic interactions (H-arene, arene-arene, greasy amino acid residues) played a significant role in the stability of the binding pose. Finally, replacing the glucosyl group with the rhamonosyl group, Kaempferol 3-O-α-rhamnoside, led to a more stable binding pose (S = −9.69 kcal/mol) in which the rhanonosyl moiety played a critical role. As shown in (Figure 2F), Kaempferol 3-O-α-rhamnoside exhibited a set of hydrophilic interactions with five amino acid residues (Glu166, Thr26, Cys145, Met165, Asn142), combined with a different type of hydrophobic interaction with different amino acid residues (H-arene, arene-arene, greasy amino acids). These results indicate that the 2-phenyl flavone scaffold could be a lead scaffold for developing a potential inhibitor against 3CL-protease activity and that the introduction of a sugar moiety at 3-position has a beneficial effect in improving the binding activity. Based on our results, Quercetin 3-O-β-glucoside has the highest binding affinity score with the ability to form stable hydrophilic and hydrophobic interactions with the binding pocket of 3CL-protease. Similarly, both Kaempferol 3-O-β-glucoside and Kaempferol 3-O-α-rhamnoside displayed considerably high binding scores. Taken together, our molecular modeling study suggested that the extract of *P. lentiscus* bark could be a beneficial source for novel flavonoid glycosides inhibitors against SARS-CoV-2 infection [68,69]. Further studies should be performed in the future to deeply explore and confirm the inhibitory activity of Quercetin 3-O-β-glucoside, Kaempferol 3-O-β-glucoside, and Kaempferol 3-O-α-rhamnoside.

## 4. Conclusions

*P. lentiscus* bark methanol extract phytochemically demonstrates the existence of carbohydrates, flavonoids, tannins, coumarins, triterpenes, and alkaloids. Deep phytochemical exploration led to the identification of methyl gallate, gallic acid, kaempferol, quercetin, kaempferol 3-O-α-rhamnoside, kaempferol 3-O-β-glucoside, and Quercetin-3-O-β-glucoside. When tested using the DPPH assay, the methanol extracts of *P. lentiscus* bark demonstrated a high free radical scavenging efficiency. The antibacterial activity of the *P. lentiscus* bark methanol extract was shown in this investigation, which might be attributable to its antioxidant properties. The antimicrobial activity is remarkable and more effective toward Gram-positive bacteria. *P. lentiscus* bark methanol extract had a potential antibacterial effect on the capabilities of *P. aeruginosa.* The *P. lentiscus* bark extract demonstrated a total antioxidant activity similar to that of ascorbic acid. Moreover, Quercetin 3-O-β-glucoside is the most effective inhibitor of SARS-CoV-2 infection, but a synergy from other constituents can work.

Furthermore, our detailed in silico molecular docking study revealed that the extract constituents have high binding affinity scores toward the 3CL-protease protein, suggesting that this extract could be used against SARS-CoV-2 infection. The industry is disposed to lower the custom of chemical additives in foodstuffs; accordingly, methanolic extract of *P. lentiscus* bark with potentially active antimicrobial characteristics could be deliberated as an expected source for the conservation or postponement of the shelf life of food. The identified chemicals are predicted to be useful in the future for the research of antibacterial agents.

## Figures and Tables

**Table 1:** Phytochemical analysis of the methanolic extract of *Pistacia lentiscus* bark.

Ingredients	*Pistacia lentiscus* Bark
Triterpenes and/or sterols	80%
Carbohydrates and/or glycosides	5.0%
Flavonoids	10%
Coumarins	2.0%
Alkaloids and/or nitrogenous compounds	3.0%
Tannins	0.5%
Saponins	-

(-) deficiency of ingredients. These percentages represent the amount of each ingredient from the total extract.

**Table 2:** The antimicrobial activity of the *P. lentiscus bark* extracts.

Microorganism	Source	*P. lentiscus* Bark Extract ^a^	Ampicillin 10 µg/disc	Gentamicin 10 µg/disc	Amphotericin 10 µg/disc
*Bacillus cereus*	Food	20 ±1.33	25 ± 1.12	NT	NT
*Salmonella paratyphi*	Food	16 ± 1.12	NT	15 ± 1.15	NT
*Saccharomyces cerevisiae*	Food	20 ± 1.22	NT	NT	10 ± 1.06
*Enterococcus feacalis*	Human	21 ± 1.03	22 ± 1.07	NT	NT
*Serratia marcescens*	Human	10 ± 1.00	NT	17 ± 1.12	NT
*Staphylococcus aureus*	Human	11 ± 1.10	25 ± 1.09	NT	NT
*Aeromonas hydrophila*	Human	20 ± 1.28	NT	17 ± 1.02	NT
*Acinetobacter baumannii*	Human	14 ± 1.05	NT	16 ± 1.09	NT
*Escherichia coli*	Human	12 ± 1.08	NT	17 ± 1.18	NT
*Klebsiella pneumoniae*	Human	12 ± 1.01	NT	15 ± 1.11	NT
*Brevundimonas vesicularis*	Human	16 ± 1.14	NT	15 ± 1.35	NT
*Pseudomonas aeruginosa*	Human	22 ± 1.80	NT	17 ± 1.48	NT
*Candida albicans*	Human	20 ± 1.09	NT	NT	10 ± 1.09

^a^ diameter (mm) of the inhibition area around the discs soaked with extract (100 µg/disc). NT: Not tested.

**Table 3:** Antioxidant activity of the methanolic extract from *Pistacia lentiscus* bark.

Plant Extract /Control	Concentration (µg/mL)	Inhibition (%)	IC_50_ Values (µg/mL)
Extract	10	32	
	50	46	54.34 ± 2.3
	100	85	
Butylated hydroxyanisole ^a^	10	28.39	
	20	57.03	17.58 ± 0.63
	30	85.11	
Ascorbic acid ^a^	10	49.76	14.73 ± 0.55
	20	67.87	

^a^ Control references.

**Table 4:** Interactions and scores of the docking process of the *P. lentiscus* bark extract (3–6) in the 3CL-protease binding pocket.

Protein	Docking Score (kcal/mol)	Interactive Residues	
		Hydrophilic Interactions	Hydrophobic Interactions
Kaempferol	−6.25	Glu166, Arg188, Asn142	Leu141, Met165, Phe140
Quercetin	−6.89	Glu166, Ser144, Gly143	Asn142, Met49, Leu141, Leu27
Quercetin 3-O-β-glucoside	−10.11	Arg188, Gln166, Asn142, Met49, Met44, Met165, His41	Val186, Phe140, Met165, Leu141
Kaempferol 3-O-β-glucoside	−9.24	Glu166, Cys145, Asn142, Thr26	His41 (arene-arene), Asn142 (H-arene), Met49, Leu27, Met165, Leu141, Phe140
Kaempferol 3-O-α-rhamnoside	−9.69	Glu166, Thr26, Cys145, Met165, Asn142	His41, Leu141, Phe140, Met49

## Data Availability

Data is contained within the article and Supplementary Materials.

## Supplementary Materials

The following supporting information can be downloaded at: https://www.mdpi.com/article/10.3390/antiox11050930/s1. NMR analysis of Pistacia bark extract.
