# Supplementary material for: Insights into the Antimicrobial, Antioxidant, Anti-SARS-CoV-2 and Cytotoxic Activities of Pistacia lentiscus Bark and Phytochemical Profile; In Silico and In Vitro Study
Source: Antioxidants (Basel). 2022 May 9;11(5):930. doi: 10.3390/antiox11050930 (PMC9138067; doi:10.3390/antiox11050930)
Supplement: Supplementary file 1 [file antioxidants-11-00930-s001.zip › antioxidants-1703312-supplementary.pdf]

Supplementary Information

Insights into the Antimicrobial, Antioxidant, Anti- SARs Cov-2 and Cytotoxic Activities of *Pistacia Lentiscus* Bark and Phytochemical Profile; *in silico* and *in vitro* study

Samy Selim [1*], Mohammed S. Almuhayawi [2], Mohanned T. Alharbi [3], Soad K. Al Jaouni [4], Afaf Alharthi [5], Basel A. Abdel-Wahab [6,7], Mervat A.R.Ibrahim[8], Amnah Mohammed Alsuhaibani [9], Mona Warrad[10], Khaled Rashed[11*]

General experimental procedures

UV/VIS: Shimadzu UV-visible recording spectrophotometer model-UV 240 (NRC, Egypt). ^{1}H-NMR and ^{13}C-NMR (Varian Unity Inova). MS (Finnigan MAT SSQ 7000, 70 ev). (Silica gel (0.063-0.200 mm for column chromatography) and Sephadex LH-20 (Pharmacia Fine Chemicals). Thin layer chromatography (TLC) F_{254} plates. Solvent mixtures, BAW (*n*-butanol:acetic acid:water 4:1:5 upper phase, 15% acetic acid: water: glacial acetic acid: 85:15). Paper Chromatography (PC) Whatman No.1 (Whatman Led.Maid Stone, Kent, England) sheets for the qualitative detection of flavonoids and sugars.

Structure Elucidation of the isolated compounds

Methyl gallate (1): 65 mg, white amorphous powder. UV λmax (MeOH): 272. 1H-NMR (DMSO-d6, 400 MHz): 6.9 (2H, s, H-2,6), 3.6 (3H, s, -OCH3). 13C-NMR (DMSO-d6, 100 MHz): 166.8 (-COO), 146 (C-3,5), 138.9 (C-4), 119.8 (C-1), 109 (C-2,6), 52 (-OCH3).

Gallic acid (2): 35 mg, white amorphous powder. UV λmax (MeOH): 270. 1H-NMR (DMSO-d6, 400 MHz): 7.1 (2H, s, H-2,6). 13C-NMR (DMSO-d6, 100 MHz): 166.9 (-COOH), 145.4 (C-3, 5), 137.8 (C-4), 121.4 (C-1), 109.6 (C-2,6).

Kaempferol (3): 8 mg, yellow powder, 1H-NMR (400 MHz, DMSO-d6): δ 8.12 (2H, d, J = 8 Hz, H-2', 6'), 6.96 (2H, d, J = 8 Hz, H-3',5'), 6.47 (1H, d, J = 2 Hz, H-8), 6.19 (1H, d, J= 2 Hz, H-6). (+) ESI-MS: m/z 287 [M+H]+.

Quercetin (4): 15 mg, yellow powder. UV λmax (MeOH): 255, 297, 371; (NaOMe): 270, 320, 420; (AlCl3): 270, 455; (AlCl3/HCl): 264, 303sh, 315sh, 428; (NaOAc): 264, 318, 383; (NaOAc/H3BO3): 259, 387. EI-MS: m/z 302.

Kaempferol 3-O-α-rhamnoside (5): 22 mg, yellow powder. 1H-NMR (400 MHz, CD3OD,): δ 7.74 (2H,d, J=8.2 Hz, H-2',6,). 6.92 (2H,d, J=8.2 Hz, H-3',5'). 6.44 (1H, d, J= 2.2 Hz, H-8), 6.24 (1H, d, J= 2.2 Hz, H-6). 5.42 (1H, d, J=2.4 Hz, H-1''), 0.94 (CH3, d, J =6.2 Hz). 13C-NMR (100 MHz, CD3OD): δ ppm 179.85 (C-4), 166.25 (C-7), 161.84 (C-5), 159.56 (C-4'), 158.28 (C-2), 136.45 (C-9), 132.28 (C-3),122.94 (C-6'), 116.84 (C-2'), 116.24 (C-3') , 106.12 (C-1'), 103.75 (C-5'), 104.74 (C-10) , 100.12 (C-1''), 95.14 (C-8), 94.92 (C-6), 73.14 (C-5''), 72.45 (C-3''), 72.34 (C-2''), 72.28 (C-4''), 17.92 (CH3-rhamnosyl).

Kaempferol 3-O-β-glucoside (6): 12 mg, yellow powder. UV λmax (MeOH): 266, 344; (NaOMe): 274, 327sh, 401; (AlCl3): 274, 302, 349, 396; (AlCl3/HCl): 274, 347, 394; (NaOAc): 274, 307, 391; (NaOAc/H3BO3): 267, 352. 1H–NMR (500 MHz, DMSO–d6,): 8.12 (2H, d, J = 8.5 Hz, H–2',6'), 6.92 (2H, d, J=8.5 Hz, H–3',5'), 6.54 (1H, d, J = 2 Hz, H–8), 6.22 (1H, d, J = 2 Hz, H–6), 5.35 (1H,d, J=7.5, H–1``), 4–3.1 (5H,m, other sugar protons).

Quercetin-3-O-β-glucoside (7): 22 mg, yellow amorphous powder: 1H-NMR (400 MHz, DMSO-d6), δ ppm 7.58 (2H, m, H-2′/6′), 6.83 (1H, d, J = 9 Hz, H-5′), 6.35 (1H,d, J = 2.5 Hz, H-8), 6.15 (1H,d, J = 2.5Hz, H-6), 5.46 (1H, d, J = 7.5 Hz, H-1′′), 3.1-4 ppm (m, sugar protons). 13C-NMR (100 MHz, DMSO-d6): δ ppm 177.83 (C-4), 162.14 (C-7), 161.73 (C-5), 157.38 (C-2), 156.89 (C-9), 149.11 (C-3′), 148.47 (C-4′), 145.38 (C-5′), 133.77 (C-3), 121.61 (C-1′), 116.65 (C-2′) 115.72 (6′),104.21 (C-10), 101.44 (C-1′′), 99.37 (C-6), 94.12 (C-8), 78.08 (C-5′′), 77.03 (C-3′′), 74.62 (C-2′′), 70.45 (C-4′′), 61.48 (C-6′′).
